# Comparative Investigation of Ultrafast Excited-State Electron Transfer in Both Polyfluorene-Graphene Carboxylate and Polyfluorene-DCB Interfaces

**DOI:** 10.3390/molecules29030634

**Published:** 2024-01-29

**Authors:** Amani A. Alsam

**Affiliations:** Department of Physical Science, College of Science, Jazan University, P.O. Box. 114, Jazan 45142, Saudi Arabia; aaalsam@jazanu.edu.sa

**Keywords:** photochemical reactions, donor-acceptor systems, time-resolved spectroscopy, organic materials, conjugated Polymers, ultrafast laser spectroscopy, photo electron transfer (PET)

## Abstract

The Photophysical properties, such as fluorescence quenching, and photoexcitation dynamics of bimolecular non-covalent systems consisting of cationic poly[(9,9-di(3,3′-N,N′-trimethyl-ammonium) propyl fluorenyl-2,7-diyl)-alt-co-(9,9-dioctyl-fluorenyl-2,7-diyl)] diiodide salt (PFN) and anionic graphene carboxylate (GC) have been discovered for the first time via steady-state and time-resolved femtosecond transient absorption (TA) spectroscopy with broadband capabilities. The steady-state fluorescence of PFN is quenched with high efficiency by the GC acceptor. Fluorescence lifetime measurements reveal that the quenching mechanism of PFN by GC is static. Here, the quenching mechanisms are well proven via the TA spectra of PFN/GC systems. For PFN/GC systems, the photo electron transfer (PET) and charge recombination (CR) processes are ultrafast (within a few tens of ps) compared to static interactions, whereas for PFN/1,4-dicyanobenzene DCB systems, the PET takes place in a few hundreds of ps (217.50 ps), suggesting a diffusion-controlled PET process. In the latter case, the PFN^+•^–DCB^−•^ radical ion pairs as the result of the PET from the PFN to DCB are clearly resolved, and they are long-lived. The slow CR process (in 30 ns time scales) suggests that PFN^+•^ and DCB^−•^ may already form separated radical ion pairs through the charge separation (CS) process, which recombine back to the initial state with a characteristic time constant of 30 ns. The advantage of the present positively charged polyfluorene used in this work is the control over the electrostatic interactions and electron transfers in non-covalent polyfluorene/quencher systems in DMSO solution.

## 1. Introduction

Conjugated polymers are attractive for many applications such as solar cell applications [1,2], optical devices [3], chemical sensors [4] and biological sensors [5,6], due to their unique properties. Among the developing conjugated polymers, conjugated polyelectrolytes (CPEs) containing a conjugated main chain and side chains with various functional groups have been intensively investigated [7,8]. Their molecular structure can easily be tuned, making them suitable for an enormous number of specific applications. By virtue of their light harvesting properties, CPEs have also been utilized as photosensitizers in fluorescent sensors [9] and solar cells applications [7,8,9,10]. The basis of such successful utilization of the CPEs in the optoelectronic applications is their chemical structures which can be easily changed as well as their semiconducting properties with large optical densities [11,12]. In addition, their high emission intensities can be also one of their important features to be used for fluorescence resonance energy transfer [13,14]. In particular, the key issues of CPEs for solar cell applications are their flexibility, along with their simple, large scale, and low-cost fabrication devices [15,16]. Another advantage is that the functional groups of the side chains can be ionic or polar moieties, which makes it easy to modify not only solubility of the CPEs in water and other polar solvents [17] but also the redox potentials, intermolecular interactions, and energy level, which determine electronic coupling [18] and the rate of electron transfer at the donor–acceptor interface [19,20,21].

It is well known that in solar cell devices, rapid electron or energy transfer to overcome electron–hole recombination is absolutely required to reach high light energy conversion efficiency [20]. In this sense, the interaction, linking, and distance between the electron donor and acceptor moieties play a crucial role. Therefore, for CPEs, in particular, they should form strong interconnections and interpenetrations with the electron acceptor moieties to attain efficient electron or energy transfer [2]. An innovative approach utilizing the electrostatic interactions between cationic CPEs with negatively charged electron acceptor moieties has consequently attracted great attention [7,19,20]. With this approach, for example, strong electrostatic interactions between cationic CPEs and DNA and DNA bases were achieved, allowing the detection of DNA and DNA bases based on the fluorescence quenching of the cationic CPEs [21]. In addition to such a fascinating method to detect DNA and DNA bases, efficient energy transfer from photoexcited cationic polyfluorene, one of the CPEs, to porphyrins demonstrated that the cationic polyfluorene can form electrostatic assembly with small molecules, and in the assembly, it acts as a photosensitizer [22].

Recently, polyfluorene with azide derivatives has been covalently linked with graphene flakes, and the produced materials have been demonstrated to have low bandgaps and high charge carrier mobility, and they are potential materials for solar cells [23]. It is therefore an interesting challenge to explore the electron or energy transfers in non-covalent polyfluorene associations, which can provide a unique study of the bimolecular electron transfer reactions of polyfluorene in the solution phase [24,25]. Like in the case of the photoexcitation dynamics of small molecules, vertical excitation of the electron donor–acceptor system would also induce electron or energy transfer [21], and the rate of the photoinduced electron transfer (PET), charges separation (CS), and charge recombination (CR) can be related to the quenching mechanism [26]. Thus, understanding the quenching dynamics of polyfluorene, as well as the electron or energy transfer from the excited polyfluorene to the quenchers, are critically important to develop solar cell materials based on water- or organic solvent-soluble polyfluorenes [27] and on solid-phase polyfluorenes [28].

In this paper, polyfluorene with positively charges, namely poly[(9,9-di(3,3′-N,N′-trimethyl-ammonium) propyl fluorenyl-2,7-diyl)-alt-co-(9,9-dioctyl-fluorenyl-2,7-diyl)] diiodide salt (PFN), that reacts with negatively charged graphene carboxylate (GC) are reported. The reasons behind using GC were because (i) it is one of strong electron acceptor moieties to several porphyrin derivatives, resulting in ultrafast and efficient electron transfer [20,29], and (ii) its opposite charge would provide strong electrostatic interactions with PFN. Because this non-covalent PFN/GC system is of interest and offers a good model of the PET in the polyfluorene electrostatically interacted with the electron acceptor, this work has been studied and scientifically reported in this paper. To make this work more interesting, a comparison between what has been discovered in this paper (PFN^+^–GC^−^) along with what has been investigated before (PFN^+^–DCB) [2] are well reported for the first time. The non-covalent associations of polyfluorene with the neutral electron acceptor 1,4-dicyanobenzene (DCB) are reported. DCB has been demonstrated as an electron acceptor in bimolecular PET in perylene/DCB systems [2]. This means that DCB is also a strong electron acceptor and may form donor–acceptor pairs with PFN. Steady-state absorption and emission spectroscopies showed the strong affinity of PFN on the GC surface and efficient quenching of the PFN fluorescence. However, by comparing the fluorescence lifetimes of PFN by GC and that by DCB shown in previous work [2], it can be clearly demonstrated that the quenching mechanism of the PFN/GC systems is static, whereas that of the PFN/DCB systems is dynamic. 

This finding is supported by the femtosecond time-resolved absorption spectra, which reveal ultrafast electron transfer from the photoexcited PFN to the GC (within 0.02 ps time scales), which is much faster than that taking place from the photoexcited PFN to the DCB (within <5 ps time scales) [2]. One of the many advantages of the present cationic polyfluorene is, therefore, the control over the electrostatic interactions and electron transfers in non-covalent polyfluorene/quencher systems in aqueous solution. Furthermore, based on polyfluorene, once it can be easily modified, the side chains of the polyfluorene in the future can be to construct new non-covalent associations.

Since the conjugated co-polymer interaction has widespread applications, and the study of photo-physics interactions between donor and acceptor systems are important even from the point of view of fundamental research, a detailed investigation of the properties of charge transfer (CT) and charge recombination (CR) in the presence of different acceptors is of considerable interest to develop insight into the behavior and applications in different fields, such as solar cells and organic photovoltaic cells.

## 2. Results and Discussion

**Figure 1:**
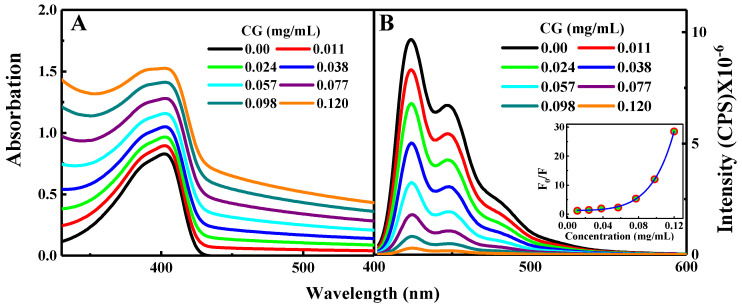
Steady-state absorption (**A**) and emission spectra (**B**) for PFN alone and PFN with GC associations. The inset gives the Stern−Volmer plot.

The absorption spectra of PFN alone and PFN with the successive addition of GC, in the absence of the GC, the absorption spectrum of PFN has a maximum peak at 402 nm with a shoulder at 383 nm and absorption cutoff at 450 nm [2,20,30]. Upon successive GC addition, the absorption spectrum of PFN is shifted upward with the GC concentration. This upward shift was demonstrated for the whole range of the recorded wavelength, and it was mainly originating from the absorption contribution of GC. The absorption and fluorescence of PFN with the successive addition of GC are shown in Figure 1A,B. 

**Figure 2:**
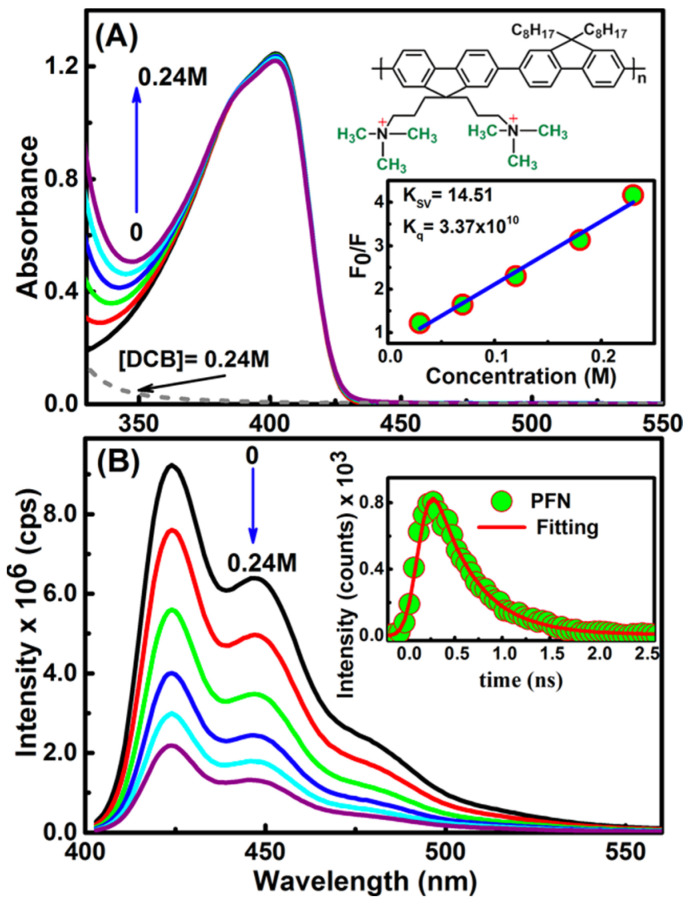
Steady-state absorption (**A**) and emission (λex = 370 nm, (**B**)) for the PFN/DCB system. The concentrations used are given in the graph. The inset gives the PFN structure, Stern−Volmer plot and the time-correlated single photon counting (TCSPC) kinetic profile collected using excitation at 370 nm of the PFN. The red line shows the fitting profile.

On the other hand, upon DCB addition, a consecutive increase was observed in the region below 360 nm, see Figure 2A [2]. This was because DCB does not absorb light in the visible region. Thus, in general, the spectra of the mixtures are the superposition of the absorptions of the PFN and the electron acceptors. This indicates that, upon the electron acceptor addition, the electronic structure of the PFN is unaffected or, in other words, the ground state interactions between the PFN and the electron acceptors do not form the CT complex.

Excitation at 380 nm shows a rise in the fluorescence spectrum of the PFN in the visible region in the range of 400–550 nm centered at 424 nm, with two vibrionic shoulders at 447 and 485 nm, respectively. Successive addition of GC results in the quenching of the PFN emission. Moreover, 97% quenching of the PFN was observed upon addition of 0.120 mg/mL GC indicating that, in comparison to a neutral DCB, a negatively charged GC was more effective for the enhanced fluorescence quenching of PFN.

**Figure 3:**
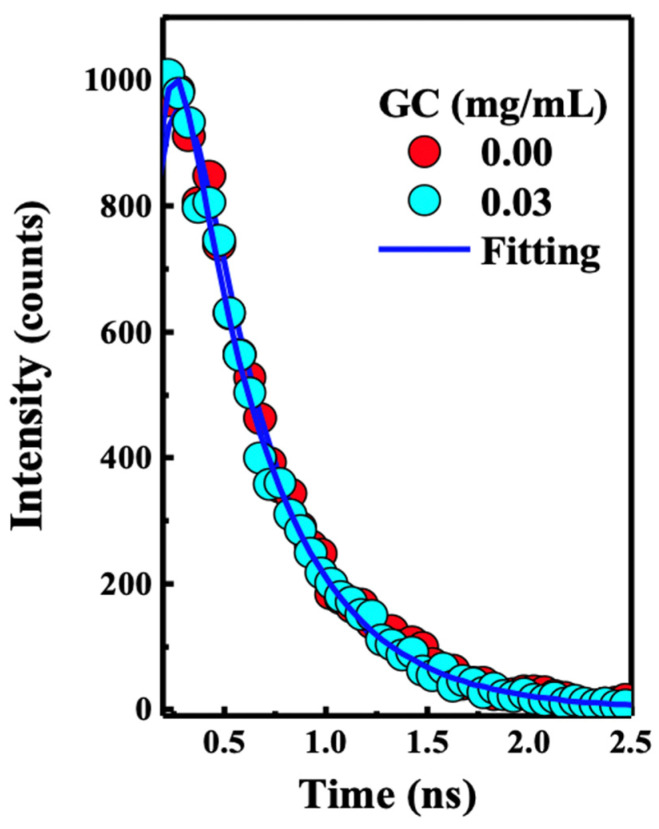
Time-correlated single photon counting (TCSPC) kinetic profile collected using excitation at 380 nm of the PFN alone (red spots), and PFN– 0.03 mg/mL GC (light blue dots). Inset section showing the fluorescence of the solution used for the TCSPC measurements. The result shows static quenching for the PFN–GC system, which agrees with the Stern–Volmer results as shown in Figure 1.

The emission spectra of the PFN and PFN–GC are shown in Figure 1B. It may be noted that the fluorescence quenching refers to the electron or energy transfer from the excited PFN to the quencher. Because the fluorescence intensity for all of the PFN peaks was decreasing at the proportional scales, resulting in the consistent spectral shape of the emission without any new emission band, here, it can be safely concluded that there is no energy transfer process from the photoexcited PFN to the electron acceptors. However, for the PFN/DCB system, see Figure 2B, in particular, saturation of the reaction mixture was observed for the DCB concentrations above 0.25 M. DCB above this saturation level leads to precipitation and a decrease in the density of the UV-vis spectrum [2]. The key difference in the fluorescence quenching behavior of the PFN by GC and DCB is revealed by the fluorescence lifetime of the PFN–GC and PFN–DCB systems at different concentrations of the quencher. In Figure 3, the mechanism of the fluorescence quenching was evaluated by monitoring the fluorescence lifetime using the TCSPC technique in the absence and presence of the graphene carboxylate (GC) quencher. It has been found that the quenching mechanism in the PFN–GC system is static, suggesting the strong electrostatic interactions facilitated by the opposite charge on PFN and GC. In the PFN–GC system, the same fluorescence lifetime of the PFN in the absence and presence of GC clearly indicates that the quenching in the PFN–GC systems proceeds according to a static mechanism. In contrast, the fluorescence lifetime of the PFN in the presence of DCB is shorter than that in the absence of DCB, supporting the dynamic nature of the interaction [2]. The different quenching mechanisms are measured to provide different rates of the PET process, where the rate of PET for quenching through static mechanism in the PFN–GC systems was found to be much faster than that in the PFN–DCB systems due to its dynamic mechanism. In addition to the driving force of the electron transfer process [31,32,33,34], in the earlier case, the rate constant depends on the electronic coupling [20], whereas in the latter case, the rate constant should be determined via the diffusion of the electron donor and acceptor moieties [35,36,37]. This issue is further evaluated by means of ultrafast TA spectroscopy, which provides detailed information on the photoexcitation dynamics. 

**Figure 4:**
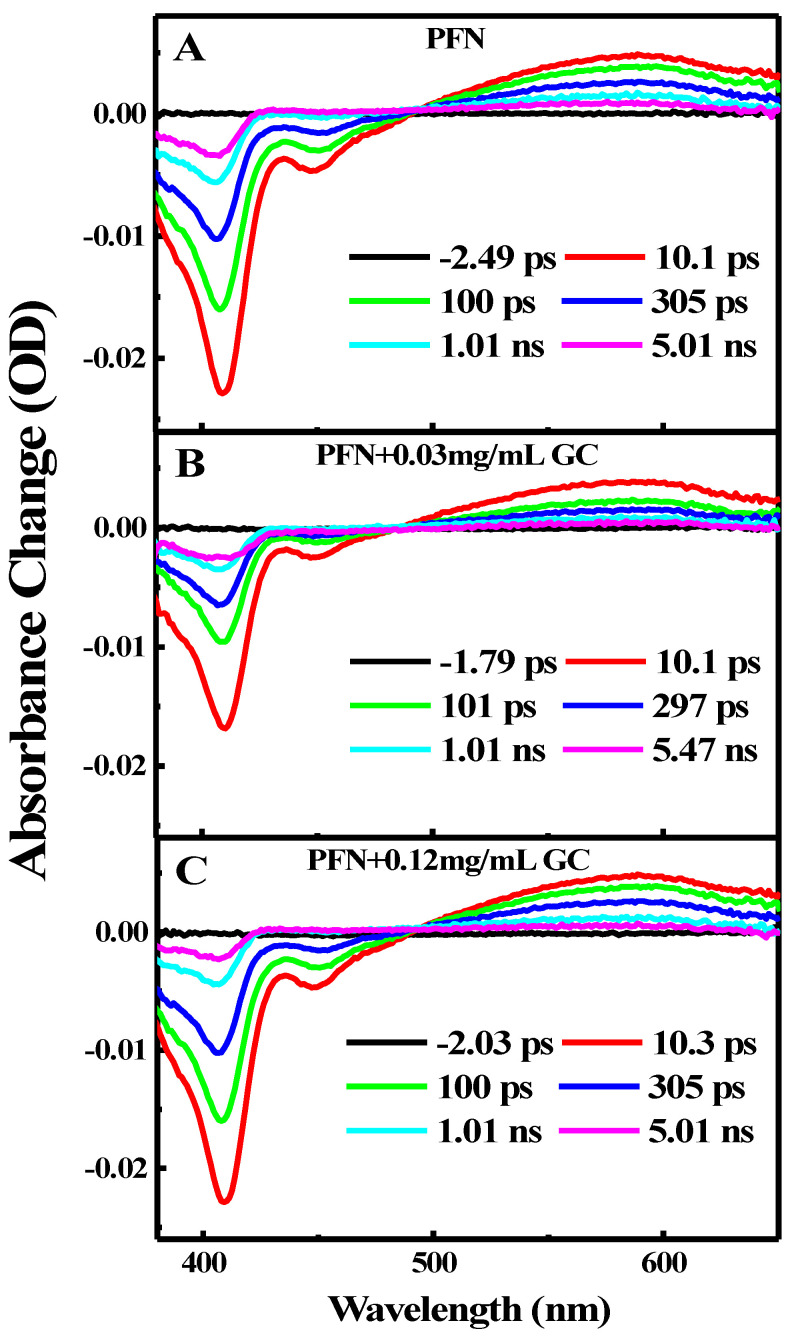
Femto-nanosecond transient absorption spectra for (**A**) PFN alone, (**B**) FPN in the presence of 0.03 mg/mL GC, and (**C**) FPN in the presence of 0.12 mg/mL GC.

Figure 4 illustrates the transient absorption TA spectra of PFN in the absence (Figure 4A) and presence of two different concentrations (0.03 and 0.12 mg/mL) of GC (Figure 4B,C) after photoexcitation at 410 nm. As shown in Figure 4A, excitation of the PFN alone immediately results in ground state bleach (GSB) at 410 nm, stimulated emission (SE) at 600 nm, and a broad excited state absorption (ESA) band centered at 580 nm. Both the GSB recovery and ESA decay are dominated by slow dynamics and the GSB is recovered on the same time scales as the ESA decay without any spectral shift and a new emerging band. Within a 5 ns time delay, the GSB is recovered up to 80% and the ESA band decays up to 70%.

**Figure 5:**
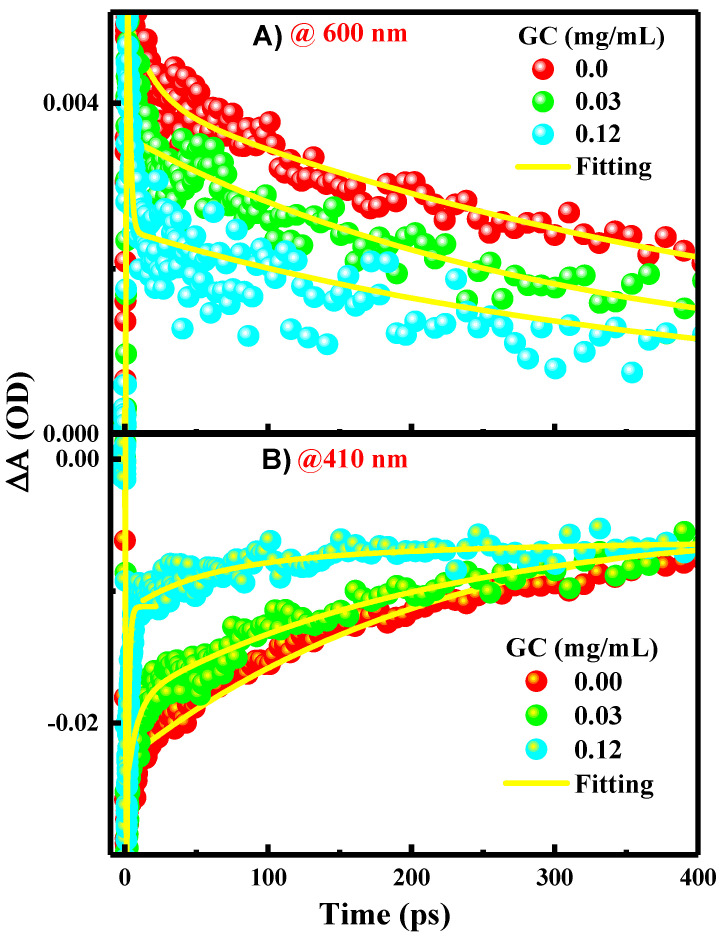
Kinetic traces extracted from femto-nanosecond TA spectra presented in Figure 4 using phtoexcitation at 600 nm (**A**), and 410 nm (**B**) for PFN in the absence and presence of 0.03 and 0.12 mg/mL GC.

As presented in the kinetics figures (Figure 5A,B), the kinetics of the GSB recovery and ESA decay can be adequately described by a single exponential fit with a time constant of 0.012 ps. This indicates that the excited single state S_1_ of the PFN has a long lifetime at a few ps time scales and it decays directly into the ground state through the CR process.

In the presence of 0.03 and 0.12 mg/mL of GC (Figure 4B,C), the transient spectral feature of the PFN–GC associations is basically similar to those of PFN alone. New absorption bands, which can be assigned to the existences of PFN^+•^–GC^−•^ radical ion pairs, are not clearly observed. However, the results shown in Figure 4A,B for the kinetics of ESA at 600 nm and GSB at 410 nm for different GC concentrations reveal that the kinetics of GSB recovery and ESA decay are GC concentration-dependent. 

The exponential fits to the data shown in the kinetic traces figure (Figure 5) propose that the kinetics of the ESA decay absolved at 600 nm (A), and GSB recovery, absolved at 410 nm (B), are biexponential with two-time constants of ~00.27 ± 00.019 ps and ~00.50 ± 00.020 ps, respectively. The rapid GSB recovery and ESA decay of the PFN in a few ps may apparently be due to the contribution from the ultrafast PET from the excited PFN to GC as well as CR recovering the PFN ground state. This thought is supported by the inspection of the kinetics in Figure 5, in which the amplitude of the fast component increases systematically with the GC concentration (80 and 70% for 0.03 and 0.12 mg/mL GC addition, respectively). The ultrafast PET and CR processes indicate the strong interactions and electronic couplings of PFN on the GC surface. Since the CR process is ultrafast, it is the reason behind the absence of PFN^+•^–GC^−•^ radical ion pairs in the spectra. This is also consistent with the trend of the static mechanism of the fluorescence quenching. In comparison with other bimolecular systems based on the electrostatic interactions of GC, it has been found that the rate of the PET process from the PFN to GC is in agreement with those observed in porphyrins/GC systems (within few tens of ps). Accordingly, it is believed that the slow component of the ESA decay and GSB recovery of the PFN in few hundreds of ps is related to the relaxation of free or uncomplexed PFN, which reduces subsequently in the presence of GC, as indicated by the lower amplitudes of the slow component with the GC concentration. 

The femtosecond TA spectra of PFN in the absence and presence of 0.05 and 0.34 M DCB are recorded after photoexcitation at 400 nm. [2] The features of the TA spectra are similar to those in the case of the PFN/GC systems, where the TA spectra show the GSB at 410 nm, SE at 450 nm, and broad ESA at 580 nm. Importantly, the peaks of the spectra are slightly blue shifted upon the DCB additions, and they are further blue shifted at longer time delays. The blue shift is most probably due to the spectral overlap between the GSB, SE, and broad ESA bands and the new emerging bands in the range 420–620 nm, with two peaks clearly observed at 370 and 600 nm at long time delays. The two emerging bands are the spectroscopic signatures of the PFN^+•^–DCB^−•^ radical ion pairs as the result of the PET from the excited PFN to DCB. 

Due to the spectral overlapping, the time constants of the PET cannot be extracted accurately from the TA spectra. Nevertheless, the kinetics of the absorption at 410 nm (Figure 5) reveal that the GSB recovery data follow a single exponential function with a time constant in a few hundred ps time scales related to the relaxation of free or uncomplexed PFN, as mentioned above. In comparison, the GSB recovery of PFN alone is more efficient than that in the presences of DCB, suggesting that DCB induces lower GSB recovery [2]. More importantly, as shown in Figure 5, the kinetics of the absorption at 410 nm indicates that the percentage of unrecovered GSB and, accordingly, amount of the long-lived PFN^+•^ radical cations are increased with DCB addition. These findings again support clearly the PET from the excited PFN to DCB, given that the PFN^+•^–DCB^−•^ radical ion pairs are long-lived and the CR is slow (in 7.09 ns time scales).

From previous work [2], it was presumed that PFN^+•^ and DCB^−•^ may form separated radical ion pairs through the CS process, as is observed in the case of perylene/DCB system as well (1.3 ns) [2,20].

The absence of ultrafast dynamics in the photoexcitation of the PFN/DCB system was consistent with the results of the steady-state fluorescence quenching of the DCB concentration-dependent fluorescence lifetime [2], which suggests that the quenching occurs through dynamic interactions. In order to evaluate the time constants of the PET process from the excited PFN to nature DCB, the rate of the PET fluorescence lifetime was extracted by fitting with a single exponential decay function. From the fluorescence lifetimes of PFN in the absence and presence of 0.24 M DCB, being 3.58 and 30.14 ns, respectively [2], the rate of the PET was estimated to be ≈2 × 10^−2^ s^−1^. This rate is much slower than that of the PET from excited perylene to DCB (in 250 ps) [2,20], and it is also slower than from PFN to GC (within 0.012 ps), the diffusion rate of typical small molecules such as DCB (in the order of 10^9^ s^−1^M^−1^), supporting the suggested diffusion-controlled PET process. 

Finally, it is noteworthy that the PET events are the reduction of the strong electron acceptor moieties by PFN. It can be considered that PET events are facilitated by the energy-level alignment between the PFN and GC to induce favorable energetics for the charge transfer process. However it is important to note that although the reduction potential of GC (−1.02 V vs. SCE) [20] is lower than that of DCB (−1.64 V vs. SCE) [2], relating to the smaller driving force from PFN to GC, the PET from PFN to GC is much faster than that from PFN to DCB, and this is due to the opposite charge on GC to achieve strong electrostatic interactions, enhancing the electronic coupling and the rate of the PET process between PFN and GC because of the close distance of the electron donor and acceptor [16,20]. Via the electrostatic interactions, one thus can control the rate of the ultrafast PET in the non-covalent associations of the cationic polyfluorene.

## 3. Experimental Section

### 3.1. Materials

Commercially available PFN (99%) was purchased from Solaris Chem Inc., Vaudreuil-Dorion, QC, Canada, and GC from ACS Materials. In all the experiments, high-purity dimethyl sulfoxide (DMSO) (99.9%; Sigma-Aldrich, St. Louis, MO, USA) was used as a solvent. All the chemicals were used without further purification. The PFN/GC mixers were prepared at standard conditions of pressure (1 atm), and temperature, (25 °C), in DMSO.

### 3.2. Steady-State Measurements

A rectangular quartz cell with a 1 cm optical path was used to measure the steady-state absorption and emission spectra of the mixtures of PFN in the presence of different concentrations of GC. The absorption spectra were recorded on a Cary5000 UV-visible spectrometer (Agilent Technologies, Santa Clara, CA, USA), and the emission spectra were collected on a Fluoromax-4 spectrofluorometer (Horiba Scientific, Piscataway, NJ, USA). 

For each experiment, a fixed volume (3 mL) of the starting solution of PFN in DMSO was put in the cell, and aliquots of the quencher dissolved in DMSO and mixed with PFN were added consecutively. The concentration of PFN was held constant at an optical density (OD) of 0.8 and 1.2 for PFN/GC and PFN/DCB systems, respectively. For the PFN/GC system, GC (in the range of 0–0.12 mg mL^−1^) was added in a constant concentration of PFN. Accordingly, for the PFN/DCB system, DCB in the range of 0–0.28 M was successively added to the PFN solution. 

The emission spectra were collected with the excitation wavelength at 380 nm. Both the entrance and the exit slits of the spectrofluorometer were kept the same (380 nm) for all the experiments. The quenching mechanism was further analyzed by monitoring the fluorescence lifetime of the PFN in the absence and in the presence of the quenchers using the time-correlated single photon counting (TCSPC) technique [2].

### 3.3. TCSPC Setup

In this setup, the excitation source was fs pulses at 370 nm (a few J of pulse energy) generated from an optical parametric amplifier (Newport Spectra-Physics, Darmstadt. Germany). The emission at 90° geometry was collected at magic angle polarization and detected using a Halcyone MC multichannel fluorescence up-conversion spectrometer with a temporal resolution of 120 fs (Ultrafast System, Sarasota, FL, USA) integrated into our existing laser system [2].

### 3.4. Femtosecond Broadband Transient Absorption (TA) Spectroscopy

Femtosecond broadband TA spectroscopy was employed to monitor the photoexcitation dynamics, including the PET, CS, and CR, in both the PFN/GC and PFN/DCB systems after excitation at 380 nm. The experimental setup of the TA spectroscopy has been previously reported in detail [2,7,20]. Briefly, the setup consists of a white-light continuum probe pulses generated by a 2 mm thick sapphire plate and spectrally tunable pump fs pulses (240–2600 nm; a few J of pulse energy) generated in an optical parametric amplifier (Newport Spectra-Physics). The pump and probe pulses were overlapped within a 2 mm thick cuvette cell containing PFN (0.4 OD) in the absence and presence of GC (0.03 and 0.12 mg/mL). In order to cover the transient spectra from a few hundred fs to s time scales, a Helios and an EOS detection system were employed with time resolutions of 120 fs and 200 ps and detection limits of 5 ns and 1 s, respectively [2]. 

## 4. Conclusions

In conclusion, I have investigated for the first time the photophysical comparison time-resolved spectroscopy between two bimolecular donor–acceptor systems. One is the positively charged PFN and negatively charged GC containing carboxylate groups in DMSO, and the other system is the interactions of the same PFN polymer and a different neutral DCB acceptor that were also investigated by the same methods, via the steady-state absorption and fluorescence spectra as well as time-resolved spectroscopy. For comparison, the mechanism of the fluorescence quenching was evaluated by monitoring the fluorescence lifetime using the TCSPC technique in the absence and presence of the graphene carboxylate (GC) quencher. It has been found that the quenching mechanism in the PFN–GC system is static, suggesting the strong electrostatic interactions facilitated by the opposite charge on the PFN and GC. This is supported by the dynamic quenching mechanism of the PFN/DCB system [2]. The photoexcitation dynamics of the PFN–GC system were further investigated using femtosecond broadband TA spectroscopy. The TA absorption spectra of PFN in the absence and presence of different GC concentrations revealed the ultrafast PET and CR processes (within few tens of ps) in the non-covalent PFN–GC systems. This finding is consistent with the trend of the static mechanism of the fluorescence quenching. In comparison, the PFN–GC systems, PET process is in ~27&196 ps time scales, suggesting a diffusion-controlled PET process. The PFN^+•^–DCB^−•^ radical ion pairs are clearly resolved, and they are long-lived. The slow CR process (in ~30 ns time scales) suggests PFN^+•^ and DCB^−•^ may form separated radical ion pairs through the CS process, which recombined back to the initial state with a characteristic time constant of 30 ns [2].

## Data Availability

Data are contained within the article.
